# Transthyretin expression in the postischemic brain

**DOI:** 10.1371/journal.pone.0221555

**Published:** 2019-09-03

**Authors:** Daniela Talhada, Isabel Gonçalves, Cecília Reis Santos, Karsten Ruscher

**Affiliations:** 1 Laboratory for Experimental Brain Research, Division of Neurosurgery, Department of Clinical Sciences, Lund University, Lund, Sweden; 2 CICS-UBI-Health Sciences Research Centre, Faculdade de Ciências da Saúde, Universidade da Beira Interior, Covilhã, Portugal; 3 LUBIN Lab—Lunds Laboratorium för Neurokirurgisk Hjärnskadeforskning, Division of Neurosurgery, Department of Clinical Sciences, Lund University, Lund, Sweden; University of Warwick, UNITED KINGDOM

## Abstract

The unknown role of the carrier protein transthyretin (TTR) in mechanisms of functional recovery in the postischemic brain prompted us to study its expression following experimental stroke. Male C57/B6 mice (age 9 to 10 weeks) were subjected to permanent focal ischemia induced by photothrombosis (PT) and brain tissues were analyzed for *ttr* expression and TTR levels at 24 hours, 48 hours, 7 days and 14 days following the insult by RT-PCR, Western blot and immunohistochemistry. Fourteen days after PT, non-specific TTR-like immunoreactive globules were found in the ischemic core and surrounding peri-infarct region by immunohistochemistry that could not be allocated to DAPI positive cells. No TTR immunoreactivity was found when stainings were performed with markers for neurons (Neuronal Nuclei, NeuN), reactive astrocytes (glial fibrillary acidic protein, GFAP) or microglia (cluster of differentiation 68, CD68). In addition, we could not find TTR by immunoblotting in protein extracts obtained from the ischemic territory nor *ttr* expression by RT-PCR at all time points following PT. In all experiments, *ttr* expression in the choroid plexus and TTR in the mouse serum served as positive controls and recombinant legumain peptide as negative control. Together, our results indicate that TTR is not synthesized in brain resident cells in the ischemic infarct core and adjacent peri-infarct area. Thus, it seems unlikely that *in situ* synthesized TTR is involved in mechanisms of tissue reorganization during the first 14 days following PT.

## 1. Introduction

Transthyretin (TTR) is a 55 kDa homotetrameric protein, composed of four identical subunits, each containing 127 amino acids [1]. TTR is mainly synthesized by the liver and by the epithelial cells of the choroid plexus (CP), which are the sources of TTR in plasma and cerebrospinal fluid (CSF), respectively [2,3]. TTR is one of the most abundant proteins in the CSF (up to 25% of total CSF protein) and, of both humans and rodents, it is the carrier protein of 3,5,3’,5’-tetraiodo-L-thyronine (T_4_) and 3,5,3’-triodo-L-thyronine (T_3_), although TTR has much higher affinity for T_4_ than T_3_.

TTR mRNA expression is usually described as being restricted to the CP and meninges, being totally absent from the hippocampus, cerebellum or cerebral cortex, in wildtype mice, shown by *in situ* hybridization and Northern blot analyses as well as RT-PCR of microdissected tissue from different brain regions and RNase protection assay [4–7]. Other studies have demonstrated that neurons express TTR mRNA and have the capacity to synthesize the protein in cortical [8] and in cerebellar neurons [9]. However, how TTR appears in brain areas other than its site of synthesis and secretion, remains to be investigated.

Based on the possibility that specific pathologies may induce the expression of TTR in different brain regions, some *in vitro* and *in vivo* studies have been conducted to assess a potential neuroprotective role of TTR in brain ischemia [10–12] and Alzheimer´s disease. In particular in Alzheimer´s disease, TTR may have the capacity to sequester amyloid beta (Aβ) [13–15]. Moreover, TTR synthesis by neurons from cortex and hippocampus has been interpreted as a natural neuroprotective response in Alzheimer´s disease [16–18]. Hence, further studies need to clarify if TTR is upregulated in the hippocampus and cerebellum [19] or if it cannot been found in the cerebral cortex, hippocampus and cerebellum in models of Alzheimer´s disease [4]. After stroke, TTR immunopositive cells were found in the ischemic territory 24 hours after permanent middle cerebral artery occlusion (MCAO) and migration of TTR from CSF has been suggested [11,12]. On the other hand, it was also shown that in the acute phase of ischemic stroke, TTR production by CP cells is upregulated, but absent in brain tissue [10].

The unknown role of TTR in recovery processes after stroke prompted us to study its expression in the post-ischemic brain. In this study, we assessed the expression of TTR in the ischemic territory at different time points, i.e. 24 hours, 48 hours, 7 days and 14 days after photothrombosis (PT).

## 2. Material and methods

### 2.1 Experimental design and animals

All animal experiments were carried out with the approval of the Malmö-Lund Ethical Committee and followed the ARRIVE guidelines (permit no. M50/2015) (S1 Table). Animals were housed in a controlled environment with a 12:12 hour light cycle, room temperature of 22°C and food and water *ad libitum*. For this experiment, 59 male C57BL/6 mice (20 to 26 g, aged 9 to 10 weeks, purchased from Charles River) were used.

**Fig 1:**
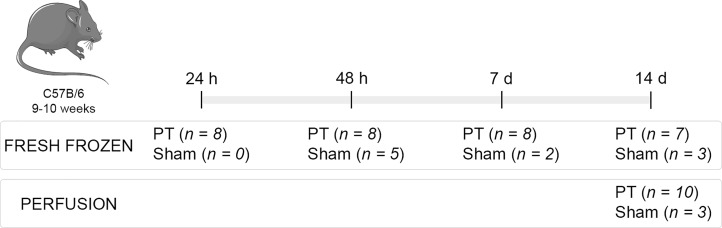
Experimental design. Brains were collected at 24 hours, 48 hours, 7 days and 14 days after brain ischemia or sham surgery to obtain protein extracts for immunoblotting. For immunohistochemistry/immunofluorescence, brains were perfusion-fixed with paraformaldehyde 4%, 14 days after PT. Abbreviation: PT—Photothrombosis.

Experimental design is described in Fig 1. For immunohistochemistry analysis, 13 animals were randomly assigned into the following groups: PT (n = 10) and Sham (n = 3). Fourteen days after the surgery, animals were perfusion fixed with 4% paraformaldehyde (PFA) and brains were collected for immunohistochemistry analysis.

To analyze TTR and *ttr* expression in the brain at different time points, 41 animals were used: PT (n = 31) and Sham (n = 10). Animals were sacrificed at 24 hours, 48 hours, 7 days and 14 days after PT or Sham surgery, as detailed in Fig 1. Animals were deeply anesthetized with pentobarbital and blood and CSF were collected. Brains were collected and immediately frozen in isopentane at −40°C (Sigma-Aldrich, Taufkirchen, Germany) and further cooled down to −70°C on dry ice. Mouse serum and CP were used as TTR positive controls and recombinant legumain (Abcam) as negative control.

Human serum samples were obtained from healthy volunteers enrolled in another study approved by the regional ethical review board of Lund University, Sweden (permit no. 2013/181).

### 2.2 Photothrombosis

Photothrombosis (PT) was carried out as described previously [20]. Briefly, animals were anesthetized with isoflurane (induction 4%, and surgery maintenance 1.5–2%) and placed into a stereotactic frame. A sagittal skin incision was made on the scalp, subcutaneous connective tissue was removed, and the skull bone was dried. Five minutes after intraperitoneal injection of the photosensitizer dye Rose Bengal (0.15 mL at 10 mg/mL; Sigma-Aldrich, Taufkirchen, Germany), the right hemisphere was illuminated with a cold light source (Schott KL 1500 LCD, intensity: 3200 K/5D) through an aperture measuring 4.0 vs 2.0 mm (equal to an illumination area of 8.0 mm^2^) for 20 minutes. During the surgery, body temperature was measured continuously through a rectal probe and maintained at 36.5 to 37.0˚C by placing the mouse on a heating pad. In total 5 animals died during surgery outside of planned euthanasia or humane endpoints. Before and after PT, temperatures and body weights were monitored throughout the experiments (S2 Table). During surgery, the animals spontaneously breathe through a face mask delivering 1.5% isoflurane in a gas mixture of 30% O_2_ and 70% N_2_O. Local analgesic bupivacaine (marcain) was subcutaneously injected 3 minutes before scalp incision. During the experiment, animals were monitored every day and did not show any signs of suffering, an there was no need for additional analgesia. Body weights were assessed every day and body temperatures were assessed before and during photothrombosis, 2 days, 7 days and 14 days after surgery.

### 2.3 Immunohistochemistry

Brain coronal sections (thickness 30 μm) from PFA perfused animals were gently washed three times in phosphate buffered saline (PBS) at room temperature (rt) and quenched with 3% H_2_O_2_ for 20 minutes. After washing (PBS 3x10 min rt), brain sections were blocked with 5% normal donkey serum (NDS) in PBS supplemented with 0.25% Triton X-100 (PBS-T) for one hour. Sections were incubated with anti-mouse polyclonal TTR primary antibody (rabbit cat# PA5-27220 Thermo Scientific Rockford, USA diluted at 1:1000), in 3% NDS PBS-T at 4°C overnight. After incubation with primary antibody, slices were washed in PBS-T (3x10 min rt) and incubated with a secondary biotinylated donkey anti-rabbit antibody (1:400 Jackson Immunoresearch, Baltimore, USA), in 3% NDS PBS-T, at room temperature for 90 minutes. Thereafter, sections were washed in PBS-T (3x10 min rt). Visualization was achieved through the Vectorstain ABC Elite kit (Vector Laboratories, CA, USA), washed in PBS in between (3x10 min rt), and using 3,3-diaminobenzidine-tetrahydrochloride (Dabsafe, Saveen Werner AB, Limhamn, Sweden), 8% NiCl_2_ and 3% H_2_O_2_. Sections were dehydrated in consecutive higher concentrations of ethanol, followed by xylene and mounted using Pertex (Histolab AB, Gothenburg, Sweden). To access specificity, primary TTR antibody was pre-incubated with TTR blocking peptide (cat# SBP3500378, Sigma-Aldrich, Saint Louis, USA), during 30 minutes at room temperature.

### 2.4 Immunofluorescence

Brain coronal sections (thickness 30 μm) from perfused animals were gently washed three times in PBS and blocked with 5% NDS in PBS-T for one hour at room temperature. Sections were incubated with rabbit anti-TTR (ThermoScientific, Rockford, USA, diluted at 1:500) together with either a mouse anti neuronal nuclei (NeuN, Merck Millipore; diluted 1:1000), or rat anti CD68 (AbD Serotec; diluted 1:200–1:500), diluted in 2–3% NDS PBS-T at 4°C overnight. Monoclonal directly Cy3 conjugated anti-glial fibrillary acidic protein (GFAP) was used to stain astrocytes (diluted at 1:5000, Sigma-Aldrich, St Louis, USA) in 3% NDS PBS-T for one hour at room temperature. After incubation with primary TTR antibody, sections were rinsed and incubated with a secondary biotinylated donkey anti rabbit antibody (Jackson Immunoresearch, Baltimore, USA) and secondary donkey anti-mouse and anti-rat antibodies conjugated with fluorescent dye Cy3 (Jackson Immunoresearch, UK), all diluted at 1:400 in 3% NDS PBS-T at room temperature for 90 minutes. Thereafter, sections were incubated with an Alexa 488 Streptavidin conjugate (Jackson Immunoresearch, UK, diluted at 1:400), for one hour at room temperature. Thereafter, sections were incubated with DAPI (final concentration 0.5 μg/ml) for 5 minutes at room temperature. Co-stainings were visualized using a confocal microscopy system (LSM510 Zeiss, Jena, Germany) and the AIM LSM 4.2 software (Zeiss).

### 2.5 Western blotting

#### 2.5.1 Brain Sample Preparation

Fresh frozen brains were placed into a brain matrix and cut, 2.2 mm to -2.2 mm relatively to bregma. For each four mm thick section, the infarct core (IC) and peri-infarct (PI) area in the ipsilateral (IPS) region were collected. The same brain regions were collected from sham operated animals. All the procedures were performed in a glove chamber at -20°C.

#### 2.5.2 Preparation of protein extracts from brain

Brain tissue was mechanically homogenized by a Dounce homogenizer in lysis buffer containing 20 mM Tris (pH 7.5), 150 mM NaCl, 1 mM EDTA, 1 mM EGTA, 2.5 mM sodium pyrophosphate, 1 mM β-glycerolphosphate, 1 mM sodium orthovanadate (Na_3_VO_4_), 1 mM phenylmethanesulfonyl fluoride (PMSF), 1% Triton-X100 and supplemented with protease inhibitor cocktail (Sigma-Aldrich, Deisenhofen, Germany). The homogenates were centrifuged 14000xg at 4°C for 20 minutes, after 20 minutes of incubation on ice. The supernatant was collected and stored at -20°C for further analysis. Total protein concentrations were determined with the Bradford assay using bovine serum albumin (BSA) as standard.

#### 2.5.3 Immunoblotting

Samples were diluted in dodecyl sulfate sodium (SDS) sample buffer and proteins denatured at 100°C for 15 minutes. Proteins were separated on a 15% SDS polyacrylamide gel. After transferring proteins onto polyvinylidene difluoride (PVDF) membranes, blocking was accomplished by incubation of the membranes in a 5% non-fat dry milk solution in Tris-Buffered Saline supplemented with 0.1% Tween (TBS-T). Following blocking, membranes were incubated with polyclonal TTR primary antibodies (rabbit cat# PA5-27220 diluted at 1:3000, ThermoScientific Rockford, USA; chicken cat# SAB3500378 diluted at 1:3000, Sigma-Aldrich) in 5% BSA (cat# A4503, Sigma-Aldrich) in TBS-T at 4°C overnight. The membranes were incubated with a secondary antibody anti-rabbit horse radish peroxidase (HRP) conjugated (diluted 1:15000, Sigma-Aldrich, Germany) in 5% non-fat dry milk solution for one hour at room temperature, or secondary biotinylated donkey anti-chicken (diluted at 1:80000), followed by anti-biotin HRP conjugated (diluted at 1:3000, Cell Signaling, USA), in 5% non-fat dry milk solution, for one hour at room temperature. To assess specificity, primary TTR antibody was pre-incubated with TTR blocking peptide (cat# SBP3500378, Merck Sigma-Aldrich, Saint Louis, USA), for 60 minutes at room temperature. The signals were visualized using a chemiluminiscence kit (Merck Millipore, Darmstadt, Germany) and charge-coupled device camera (Fujifilm LAS 1000, Fujifilm, Tokyo, Japan). Membranes were stripped and reprobed with anti β-actin HRP conjugated (diluted at 1:50000, Sigma-Aldrich, Deisenhofen, Germany).

### 2.6. mRNA quantification by Real-time PCR

Brain tissue samples and CP were homogenized in RNeasy mRNA kit (Qiagen) and total RNA extraction was performed according to the manufacturers protocol. Synthesis of cDNA was carried out using the Scrip cDNA Synthesis Kit (Bio-Rad). cDNA was analyzed using real-time PCR SsoAdvance SYBR Green Supermix from Bio-Rad using appropriate primers (sequences: fw 95 –GGTGCTGGAGAATCCAA; rev 345 –CATCCGCGAATTCATGGA) and run on a Bio-Rad CFX96 real-time quantitative PCR (qPCR) system. Reactions were performed using 0.2 μM of each primer and 1 μL of diluted cDNA (1:20) in a final volume of 10 μL. Quantification cycle threshold (Cq = 39) values per target were manually estimated. Gene expression was normalized to the housekeeping gene GAPDH and calculated using the 2^−ΔCt^ method. Melt curve analyses were performed to ensure the specificity of qPCR products. All assays included at least two biological replicates with two technical replicates each, positive control and a non-template control.

## 3. Results

### 3.1 Transthyretin expression in the ischemic territory 14 days after permanent stroke

**Fig 2:**
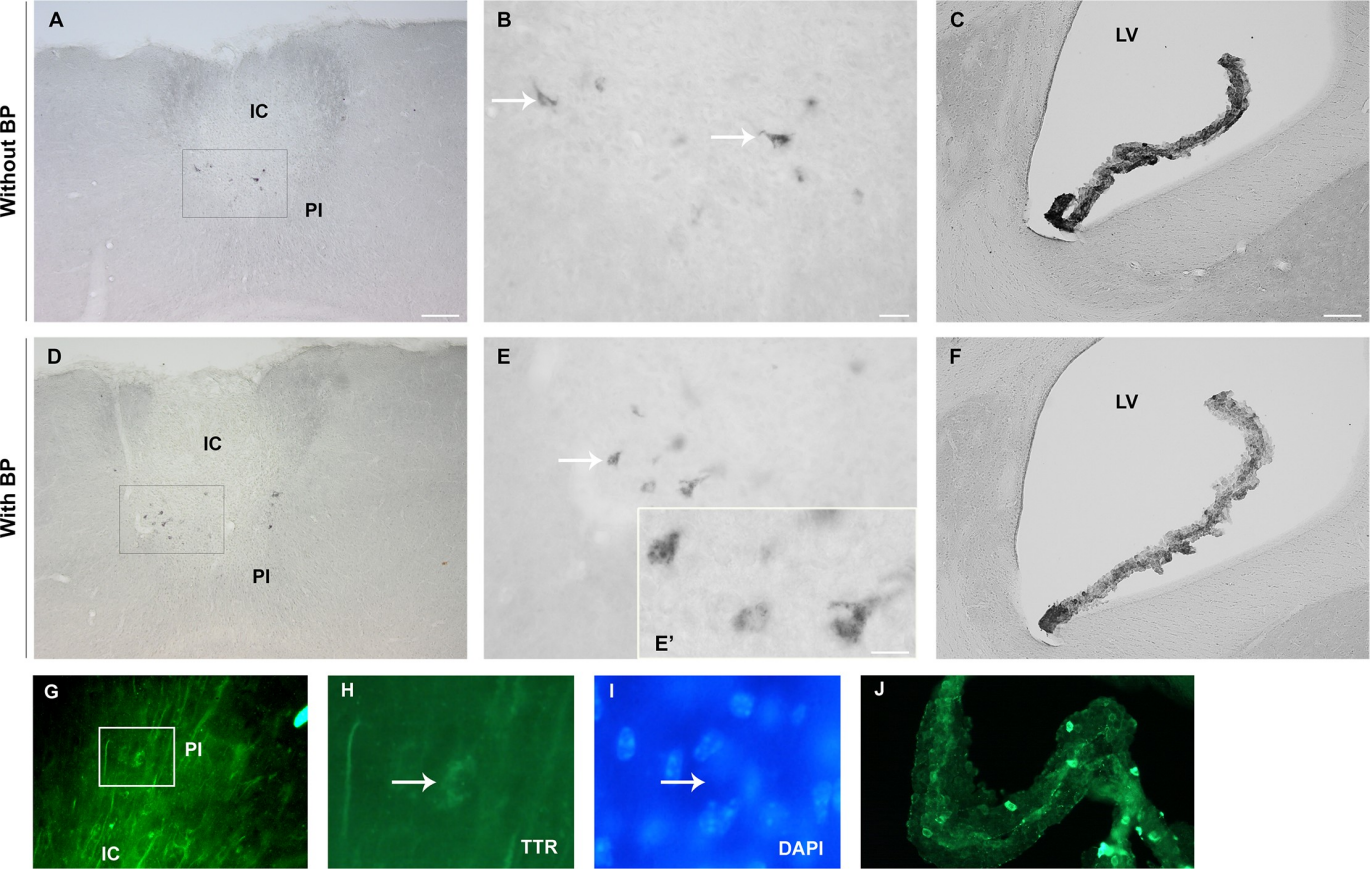
Immunohistochemistry in the postischemic brain. Scattered cells were immunoreactive for TTR in the IC and PI 14 days after ischemic stroke. Low magnification overview of the ischemic territory using 1:1000 anti-TTR rabbit antibody (ThermoScientific cat# PA5-27220, Rockford, USA) **(A)** without and **(D)** with BP. **(B, E and E´)** Higher magnification micrographs of images (A) showing cells immunoreactivity for TTR (white arrows). **(C)** Epithelial cells from CP were positive for TTR and **(F)** partially blocked with BP. **(G)** Staining for TTR (green, AF488) and with higher magnification in (J) together with DAPI (H). **(J)** Epithelial cells from CP from the same section. Scale bars: **A**, **C**, **D** and **F—**100 μm, **B** and **E—**20 μm and **E´** - 10 μm. BP–Blocking peptide; CP—Choroid plexus; IC—Infarct core; PI–Peri-infarct area; TTR–Transthyretin; LV–Lateral ventricle.

We performed immunohistochemistry analysis to evaluate if cells express TTR in the postischemic brain, i.e the infarct core (IC) and peri-infarct (PI) area 7 and 14 days after PT. As shown in Fig 2A and 2B we found some scattered TTR-like immunoreactive globules in the IC and in the PI area, however, their appearance was inconsistent and immunoreactivity (ir) was not found in all animals subjected to PT. Globules showed irregular morphology, mostly in a triangular shape and TTR appeared in granules indicative of dying cells. In addition, ir could not be allocated to DAPI (Fig 2G to 2J). Dilution of primary antibody revealed a slight reduction of this unspecific ir. Importantly, these signals could not be blocked by a specific TTR blocking peptide (Fig 2D and 2E) while signals in cells positive for TTR in the CP were partially blocked (Fig 2F). In Sham operated animals, TTR was only found in the CP.

### 3.2 Phenotyping of Transthyretin expressing cells in the ischemic territory

**Fig 3:**
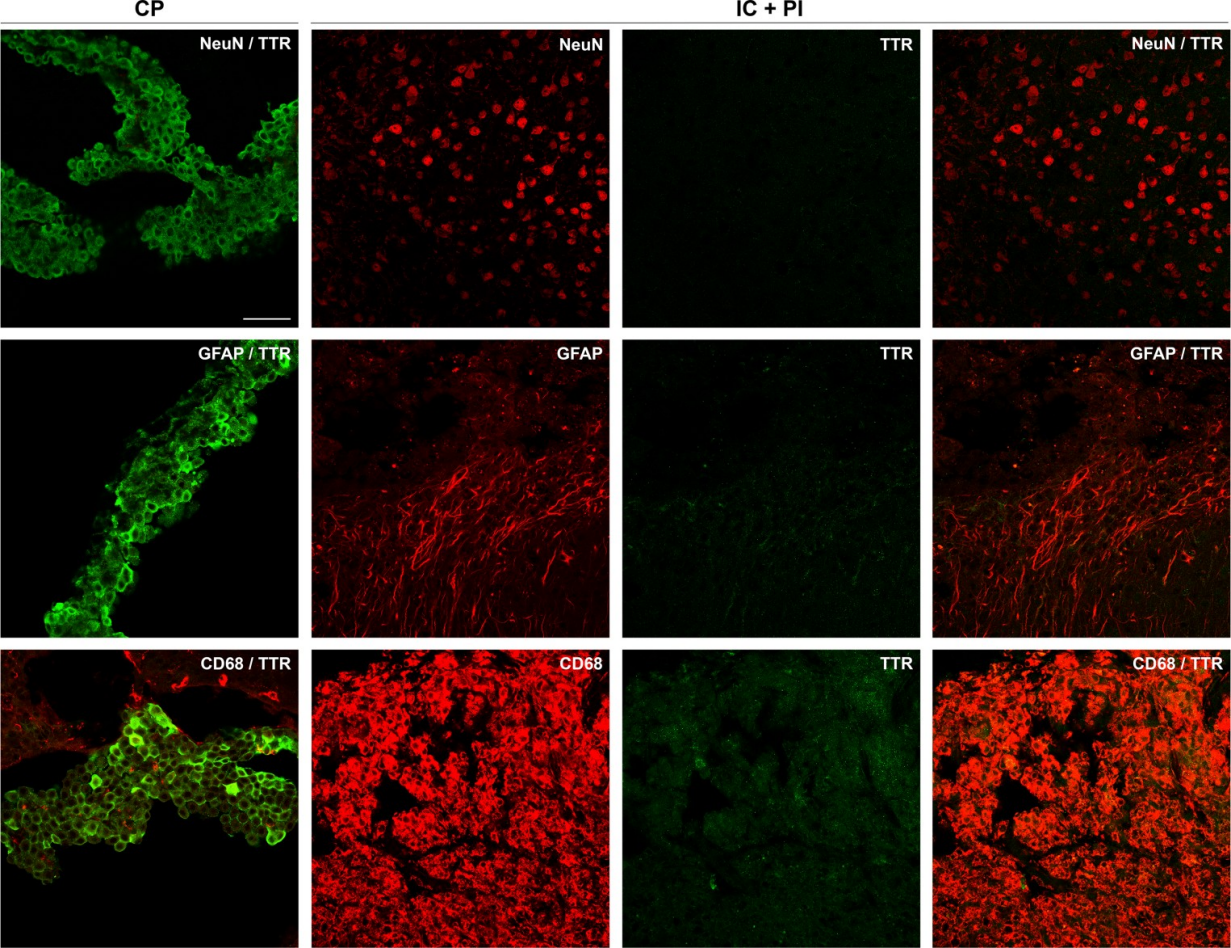
Phenotyping of TTR positive cells. TTR immunoreactivity (AF488, green) was not found in NeuN positive neurons, GFAP positive astrocytes or CD68 positive microglia/macrophages, 14 days after PT. NeuN, GFAP or CD68 are shown in red (Cy3). TTR is expressed in the epithelial cells of CP, used as a positive control. Scale bars 50 μm. Rabbit anti-TTR cat# PA5-27220 ThermoScientific Rockford, USA diluted at 1:500. CP–Choroid plexus; GFAP–Glial fibrillary acidic protein; IC–Infarct core; NeuN–Neuronal nuclei; PI–peri-infarct area; PT–Photothrombosis; TTR–Transthyretin.

To further characterize these cells, we performed immunofluorescence co-stainings with neuronal, astrocytic and microglia markers, in brain sections from animals subjected to PT. While specific TTR ir was found consistently in the CP of all animals, no specific TTR ir was detected in the ischemic territory. In particular, we did not observe TTR ir in NeuN positive neurons and GFAP positive reactive astrocytes in the proximal peri-infarct area. Also, CD68 positive microglia/macrophages mainly accumulating in the IC did not show TTR staining (Fig 3).

### 3.3 Transthyretin levels in the ischemic territory

**Fig 4:**
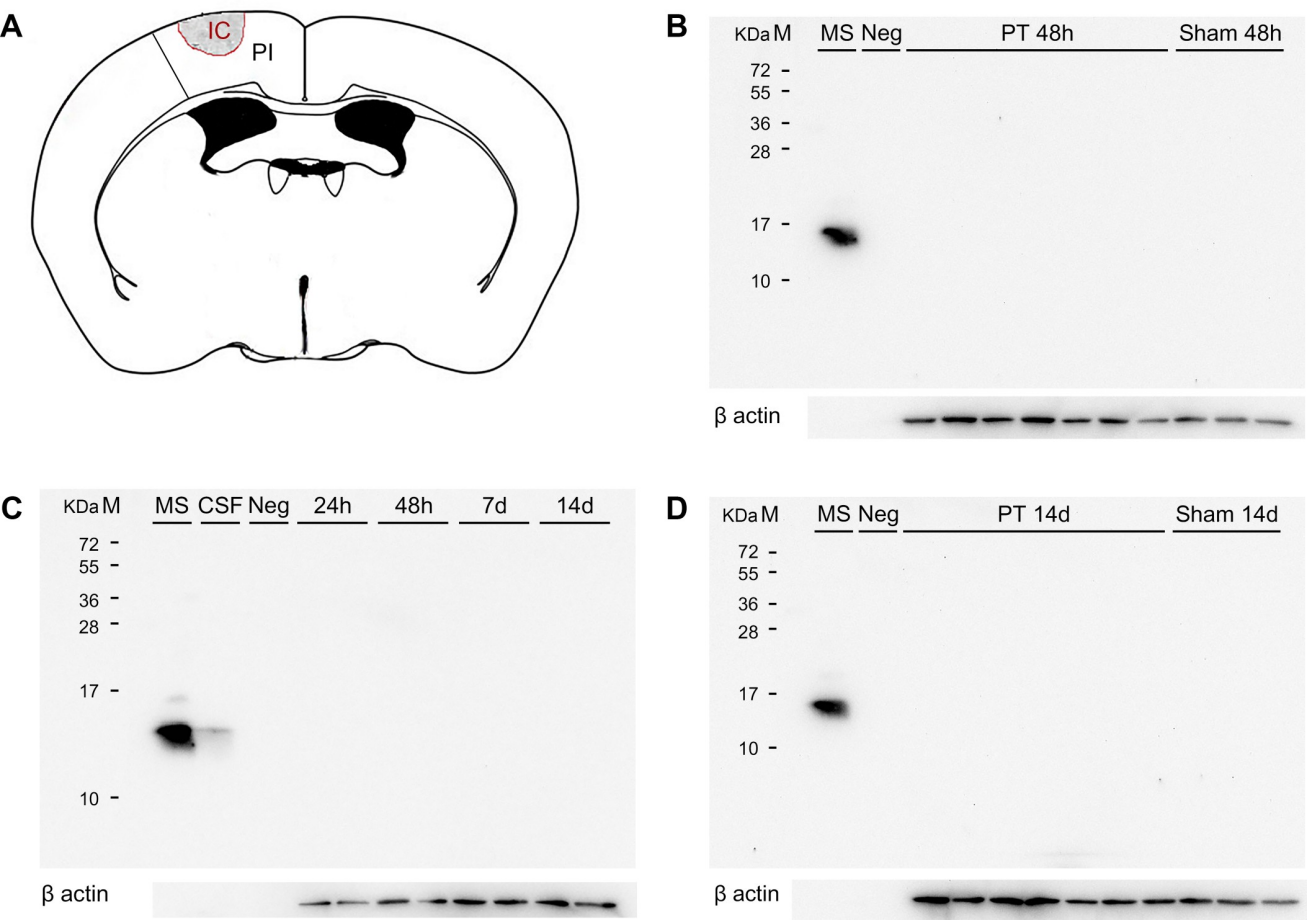
TTR levels at different time points after PT. **(A)** The ischemic territory region was sampled for protein extraction (coronal section of an adult mouse brain adapted from [21]. **(B)** TTR protein is absent in the ischemic territory 48 hours after PT and absent in the brain parenchyma of wild type mice not subjected to ischemic stroke. **(C)** TTR is not expressed in the ischemic territory at different time points after PT: 24 hours, 48 hours, 7 days and 14 days, respectively. **(D)** TTR protein is absent in the ischemic territory 14 days after PT and absent in the brain parenchyma of sham operated mice. Loading of 10 μg of total protein. Anti-TTR rabbit antibody diluted at 1:3000 (ThermoScientific cat# PA5-27220, Rockford, USA). CSF–cerebrospinal fluid; IC—Infarct Core; M–Marker; Neg–negative control (recombinant legumain 2 μg); PI–Peri-infarct area; MS–Positive control (mouse serum); PT–Photothrombosis; TTR–Transthyretin.

To further evaluate the presence of TTR in the ischemic territory at different time points after stroke, protein extracts from the ischemic territory (peri-infarct and infarct core border) were used for Western blotting (Fig 4A). Samples from mice subjected to PT and sham operated did not show a specific 15 kDa TTR band at 24 hours, 48 hours, 7 days and 14 days after PT corresponding to monomeric or tetrameric TTR, respectively. Likewise, no signals were detected in the negative control. In contrast, mouse serum and mouse CSF showed a specific 15 kDa band (Fig 4B to 4D).

**Fig 5:**
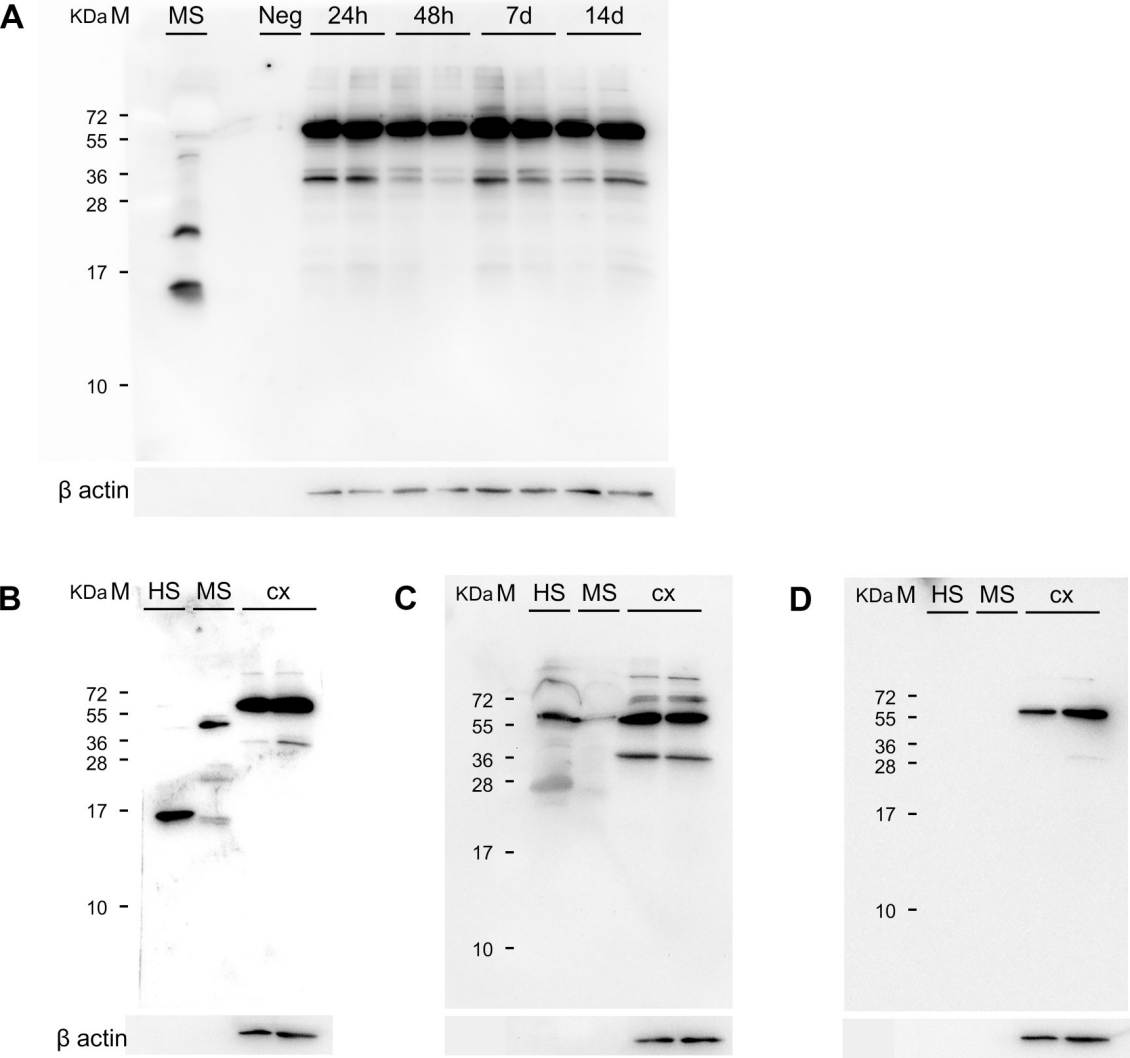
Evaluation of a chicken TTR antibody and TTR levels in brain parenchyma. **(A)** In contrast to protein extracts obtained from ischemic territory (infarct core–peri-infarct area) at 24 hours, 48 hours, 7 days and 14 days after PT, the monomeric form of TTR is found in samples of mouse serum, used as positive control. **(B)** TTR in human and mouse serum and absence of the protein in the cortical ischemic territory 14 days after PT. Instead, a band of approximately 60 to 65 kDa is found, not corresponding to the TTR protein **(C)** Blocking of specific TTR bands by preincubation with a specific TTR peptide. **(D)** Western blot performed without primary antibody incubation. HS–Human serum; M–Marker; MS–mouse serum; PT–Photothrombosis; TTR–Transthyretin; cx–cortex.

Since we essentially did not find TTR in the postischemic brain, we performed additional Western blots using a chicken anti TTR antibody. Again, we observed specific TTR bands in lanes loaded with mouse serum as well as in lanes loaded with human serum supporting the specificity of this TTR antibody. However, in contrast to Western blots using the rabbit primary antibody we obtained an additional band of approximately 60 to 65 kDa. This band could not be blocked as the 15 kDa monomer of TTR, further corroborating that this band does not correspond to the TTR tetramer (Fig 5C). Interestingly, this band is also present in absence of primary antibody suggesting a direct binding of the secondary antibody (Fig 5D). Together, only the 15 and the 55 kDa band was found to be specific for TTR. Both antibodies were recognizing the monomeric form of TTR at 15 kDa in positive controls (human and mouse serum).

**Fig 6:**
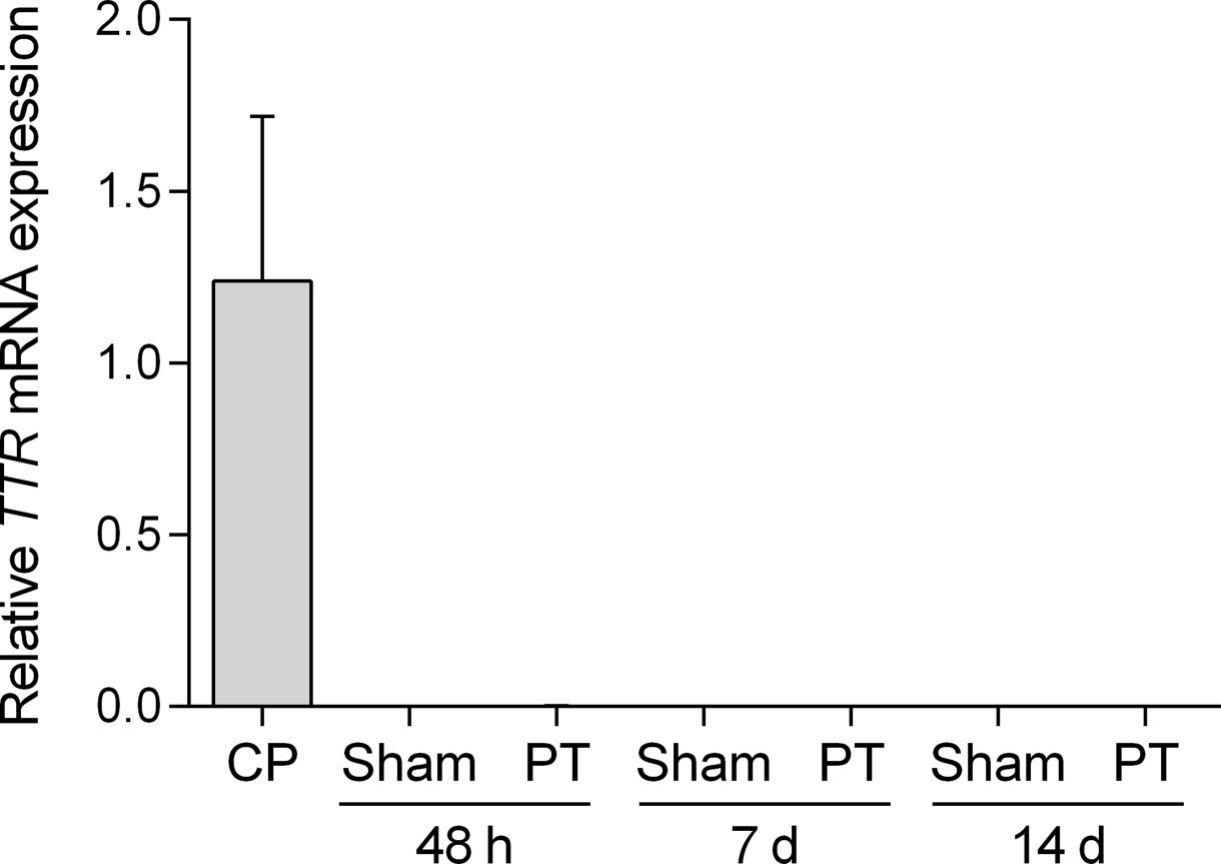
Expression of *ttr* in the postischemic brain. Relative expression *ttr* to GAPDH mRNA was semi-quantified by real time PCR in the choroid plexus (positive control) and brain parenchyma. Compared to the control, *ttr* is not expressed in brain parenchyma after photothrombosis or sham surgeries after 48 hours, 7 days and 14 days, respectively. Data represents means ± SEM of n = 2 to 5 mice. CP–Choroid Plexus; PT–Photothrombosis; TTR–Transthyretin.

To confirm negative results from immunohistochemistry and Western blot experiments, we performed quantitative RT-PCRs. As shown in Fig 6, expression of *ttr* was found in all CP samples (relative expression 1.24 ± 0.48, n = 5). In contrast, we found extremely low expression levels in samples obtained from the ischemic territory at all time points (48 h: 0.0008 ± 0.0008, n = 5; 7 d: 7.22E-06 ± 5.94E-06, n = 4; 14 d: 5.29E-05 ± 2.56E-05, n = 3) and respective sham operated mice (48 h: 7.57E-06 ± 4.17E-06, n = 3; 7 d: 7.42E-07 ± 1.64E-07, n = 2; 14 d: 6.68E-06 ± 3.30E-06, n = 2). From these experiments, we conclude that compared to CP samples *ttr* expression is not detectable in brain parenchyma. Moreover, low levels of *ttr* expression did not result in relevant protein levels of TTR in brain parenchyma.

## 4. Discussion

The present study was conducted to evaluate if transthyretin (TTR), a major transport protein for thyroid hormones and retinol-binding protein in the plasma and CSF, is expressed in the postischemic brain during the first 14 days following permanent focal ischemia induced by photothrombosis (PT). Analysis of the infarcted and peri-infarct brain tissue by immunoblotting, immunohistochemistry and RT-PCR revealed that there is no evidence for the expression of TTR in the brain parenchyma. Nevertheless, some scattered cell bodies positive for TTR were found in the ischemic infarct core and proximal peri-infarcted tissue 14 days after PT. We did not observe TTR positive neurons, any expression of the protein in astroglial cell populations or microglia/macrophages in the peri-infarct area. During the discussion, we will elaborate on possible functions of TTR in the brain and discuss results regarding the expression of TTR after stroke. Moreover, we will discuss our results showing scattered TTR positive cells in the ischemic infarct core.

Attention has been given to TTR as a neuroprotective protein, in particular in Alzheimer´s disease [13–15] and stroke [10–12] through its putative upregulation in the brain. In the context of Alzheimer´s disease, TTR has been suggested as a biological sequester of Aβ, since it is able to bind to soluble Aβ peptide, preventing the formation of amyloid fibers [22,23]. Some studies in rat and mouse models of Alzheimer´s disease show an overexpression and production of mRNA transcription of TTR in the hippocampus [19,24,25]. *In vitro* studies also support TTR synthesis in neurons as neuroprotective in Alzheimer´s disease, since primary cultures of mixed cortical and hippocampal neurons from APP23 mice express TTR [17,18], and there is an upregulation of *ttr* in the SH-SY5Y neuroblastoma cell line over-expressing APP_695_ isoform [16]. *TTR* expression has also been found in the frontal cortex of postmortem brain tissues from patients with Alzheimer´s disease [26].

After stroke, TTR immunopositive cells have been detected in the infarct core 24 h after permanent occlusion of the middle cerebral artery with no expression of *ttr* in the postischemic brain [12]. Recently, it has been reported that intranasal and intracerebroventricular delivery of an anti-TTR nanobody 169F7 reached several brain regions and spinal cord in naïve mice. From differences in the concentration of nanobodies in different regions of the CNS the authors concluded that TTR is synthesized in these tissues [27]. On the other hand, it has been suggested that the results observed in brain tissues are due to contamination by CP. TTR was not found either in the cerebral cortex, hippocampus or cerebellum, verified by TTR immunoreactivity, immunoblotting and TTR mRNA expression in wildtype mice and APP-V717I and Tg2576 mice models of Alzheimer´s disease after laser microdissection [4]. Likewise, upregulation of TTR has been shown exclusively in the CP in a proteomic study in rats subjected to transient MCAO supporting the idea that TTR expression is restricted to the CP [10]. In the present study, we found TTR synthesized in the CP but not in brain parenchyma. Our study clearly shows that TTR is not expressed in brain resident cells of the ischemic territory of C57/B6 mice, 24 hours, 48 hours, 7 days and 14 days after permanent focal ischemia. Scattered and inconsistently appearing TTR positive cells found in the ischemic territory are considered as cellular debris supported by their random morphology. Positive cells were found randomly throughout the study. Immunoreactivity was not responsive to titration of antibodies neither to a specific TTR blocking peptide. We excluded that immunoreactivity reflects a *de novo* TTR synthesis in neurons since RT-PCR experiments showed non-detectable levels of *ttr*, neither found we evidences for TTR transport from the blood or CSF. Results from our RT-PCR confirm previous results showing that *ttr* is not expressed in brain parenchyma 24 hours after permanent MCAO [12]. If TTR like proteins such as TTR-52, a bridging molecule in the phagocytic process by phagocytes and in the regeneration of axons, are expressed, remains open and might be focus of future studies. In *Caenorhabditis elegans*, a TTR-like protein mediates the recognition of apoptotic cells by crosslinking with Phosphatidyl Serine (Eat me Signal) with a receptor in the phagocyte (macrophages/microglia), being involved in the engulfment of apoptotic cells [28,29].

Nevertheless, and despite of its absence in brain parenchyma, TTR has been shown to promote neuroprotection. Activation of neuroprotective pathways is partially mediated by binding of high concentrated exogenous TTR to low density lipoprotein-related protein 2 (LRP2) in neuronal cultures. This interaction is also involved in TTR mediated neurite outgrowth. Neuronal cultures from LRP2 +/- /TTR deficient mice showed reduced neurite outgrowth compared to cell cultures from LRP2 +/+ /TTR deficient mice, both treated with TTR [11]. In contrast, TTR deficiency alone has no effect on ischemic damage in mice subjected to permanent MCAO [15]. Some immediate effects on lesion size 24 hours after stroke onset were observed in TTR deficient mice heterozygous for heat shock transcription factor 1 (HSF1) [12]. The difference in lesion size and outcome between TTR deficient mouse strains might be explained by reduced or suppressed levels of HSF1 resulting in a compromised heat shock response during the first hours after stroke onset [30]. In addition, TTR is reported as a neurogenic factor in the peripheral nervous system, enhancing neurite outgrowth *in vitro* and nerve regeneration *in vivo* studies [31,32].

## 5. Conclusions

Independent of its role as a transport protein, TTR has been studied as neuroprotective molecule in brain pathologies such as stroke. Here we found that TTR is not expressed in the infarct core and peri-infarct area during the first 14 days after photothrombosis, being restricted to the choroid plexus. Nevertheless, scattered cells showing TTR-like immunoreactivity were found in the infarct core in some animals, which may correspond to unspecific binding of the antibody to cellular debris, due to their morphology and inconsistent appearance. Although TTR is absent from brain parenchyma after stroke, it is neuroprotective *in vitro* models of ischemia, and we cannot exclude that CP derived TTR may provide similar effects *in vivo*.

## Supporting information

S1 Table The ARRIVE guidelines checklist animal research: Reporting in vivo experiments. (PDF)

S2 Table Mean of body weights and temperatures of animals over the time course of the experimental procedures. BW–Body weight; IHC–Immunohistochemistry; PT–Photothrombosis; SD–Standard deviation; Temp–Temperature; WB–Western Blotting.
(DOCX)
