# Correction: Transthyretin expression in the postischemic brain

**DOI:** 10.1371/journal.pone.0235527

**Published:** 2020-06-25

**Authors:** Daniela Talhada, Isabel Gonçalves, João Carlos Gomes, Maria João Saraiva, Cecília Reis Santos, Karsten Ruscher

João Carlos Gomes and Maria João Saraiva should be included in the author byline. João Carlos Gomes should be listed as the third author and Maria João Saraiva should be listed as the fourth author. Their affiliations are 4: Instituto de Investigação e Inovação em Saúde (I3S), University of Porto, Porto, Portugal and 5: Molecular Neurobiology Unit, IBMC—Institute for Molecular and Cell Biology, University of Porto, Portugal.

The correct citation is: Talhada D, Gonçalves I, Gomes JC, Saraiva MJ, Reis Santos C, Ruscher K (2019) Transthyretin expression in the postischemic brain. PLoS ONE 14(9): e0221555. https://doi.org/10.1371/journal.pone.0221555

The updated author contributions are as follows:

**Conceptualization:** Daniela Talhada, Karsten Ruscher

**Data curation:** Daniela Talhada

**Formal analysis:** Daniela Talhada

**Funding acquisition:** Isabel Gonçalves, Cecília Reis Santos, Karsten Ruscher

**Investigation:** Daniela Talhada, Karsten Ruscher

**Methodology:** Daniela Talhada, Karsten Ruscher

**Project administration:** Karsten Ruscher

**Resources:** Karsten Ruscher

**Supervision:** Karsten Ruscher

**Validation:** Isabel Gonçalves, Cecília Reis Santos, João Carlos Gomes, Maria João Saraiva, Karsten Ruscher

**Writing–original draft:** Daniela Talhada

**Writing–review & editing:** Isabel Gonçalves, Cecília Reis Santos, João Carlos Gomes, Maria João Saraiva, Karsten Ruscher

The raw data underlying Fig 1 and S2 Table are missing from the list of Supporting Information. The authors have provided the data as Supporting Information in S1–S3 Files. With this correction [1], all relevant data are now provided.

## Supporting information

S1 File Body weights of individual mice before and after photothrombosis (PT) or sham operation. (PDF)

S2 File Body temperatures of individual mice before and after photothrombosis (PT) or sham operation. (PDF)

S3 File Raw qRT-PCR data of individual mice after photothrombosis (PT) or sham operation and positive controls obtained from choroid plexus from five individual mice. (PDF)
