# Supplementary material for: Transthyretin expression in the postischemic brain
Source: PLoS One. 2019 Sep 3;14(9):e0221555. doi: 10.1371/journal.pone.0221555 (PMC6719853; doi:10.1371/journal.pone.0221555)
Supplement: S2 Table — BW–Body weight; IHC–Immunohistochemistry; PT–Photothrombosis; SD–Standard deviation; Temp–Temperature; WB–Western Blotting. (DOCX) [file pone.0221555.s002.docx]

| **TREATMENT GROUPS** | | | **Before** | | **24 hours** | | **48 hours** | | **7 days** | | | **14 days** | |
| --- | --- | --- | --- | --- | --- | --- | --- | --- | --- | --- | --- | --- | --- |
|  |  |  | **BW g**  **(mean ± SD)** | **Temp ºC**  **(mean ± SD)** | **BW g**  **(mean ± SD)** | **Temp ºC**  **(mean ± SD)** | **BW g**  **(mean ± SD)** | **Temp ºC**  **(mean ± SD)** | **BW g**  **(mean ± SD)** | **Temp ºC**  **(mean ± SD)** | **BW g**  **(mean ± SD)** | | **Temp ºC**  **(mean ± SD)** |
| **PT** | 24 h | WB (n = 8) | 25.2 ± 0.79 | 37.21 ± 1.39 | 23.94 ± 0.68 | 36.81 ± 0.97 | - | - | - | - | - | | - |
|  | 48 h | WB (n = 8) | 25.28 ± 0.71 | 37.31 ± 0.98 | - | - | 23.69 ± 1.18 | 37.64 ± 0.76 | - | - | - | | - |
|  | 7 d | WB (n = 8) | 25.61 ± 0.73 | 37.9 ± 0.88 | - | - | - | - | 25.78 ± 0.88 | 36.88 ± 0.31 | - | | - |
|  | 14 d | WB (n = 7) | 23.19 ± 1.47 | 37.11 ± 0.93 | 21.81 ± 1.79 | 37.33 ± 0.21 | 21.7 ± 1.68 | 37.86 ± 0.38 | 22.56 ± 2.01 | 38.2 ± 0.7 | 23.09 ± 2.24 | | 37.76 ± 0.63 |
|  |  | IHC ( n = 10) | 24.43 ± 1.91 | 37.25 ± 0.71 | 23.22 ± 1.36 | 37.74 ± 0.69 | 23.01 ± 1.2 | 37.5 ± 0.73 | 23.75 ± 1.64 | 38.02 ± 0.45 | 24.17 ± 1.49 | | 38.06 ± 0.74 |
| **Sham** | 48 h | WB (n = 5) | 26.1 ± 0.78 | 37.7 ± 1.31 | - | - | 25.08 ± 0.28 | 37.34 ± 0.45 | - | - | - | | - |
|  | 7 d | WB (n = 2) | 25.4 ± 0.14 | 38.6 ± 0.14 | - | - | - | - | 26.3 ± 0.42 | 38.15 ± 0.07 | - | | - |
|  | 14 d | WB (n = 3) | 23.53 ± 0.87 | 37.5 ± 0.35 | 22.3 ± 0.7 | 36.87 ± 0.67 | 22.17 ± 0.51 | 37.57 ± 0.55 | 23.07 ± 0.79 | 37.5 ± 0.44 | 23.17 ± 0.55 | | 37.53 ± 0.23 |
|  |  | IHC ( n = 3) | 24.57 ± 1.66 | 37.33 ± 0.9 | 24.53 ± 1.04 | 37.4 ± 0.72 | 24.4 ± 1.22 | 37.5 ± 0.26 | 24.6 ± 1.73 | 38.53 ± 0.71 | 25.5 ± 1.49 | | 38.4 ± 1.13 |

S1 Table. Body weights and temperatures of mice before and after photothrombosis or sham operation. Abbreviations: WB – Western blot, IHC – immunohistochemistry.
