# Supplementary material for: Correction: Transthyretin expression in the postischemic brain
Source: PLoS One. 2020 Jun 25;15(6):e0235527. doi: 10.1371/journal.pone.0235527 (PMC7316325; doi:10.1371/journal.pone.0235527)
Supplement: S1 File — (PDF) [file pone.0235527.s001.pdf]

Body weights (g)

PT 24 hours

animal n.	pre	d1
**61**	24.7	23.7
**62**	25.7	23.7
**63**	26.4	25.2
**64**	25.8	24.1
**65**	25.2	24.4
**94**	25.2	24
**98**	24.8	23.5
**99**	23.8	22.9

PT 48 hours

animal n.	pre	d2
**67**	25.2	23.2
**68**	25.4	22.2
**69**	25.4	23
**70**	24.3	22.3
**92**	24.2	23.9
**93**	25.7	25.1
**100**	25.7	24.9
**101**	26.3	24.9

PT 7 days

animal n.	pre	d7
**76**	26.5	25.8
**77**	25.8	27.7
**78**	25.8	24.9
**109**	25.1	25.2
**110**	26.4	25.9
**111**	26	25.9
**112**	24.6	25.8
**113**	24.7	25

Sham 48 hours

animal n.	pre	d2
**102**	26.3	25.4
**103**	26.3	25.2
**104**	27.2	25.2
**105**	25.2	24.9
**106**	25.5	24.7

Sham 7 days

animal n.	pre	d7
**107**	25.5	26.6
**108**	25.3	26

Body weights (g)

PT 14 days

animal n.	pre	d1	d2	d3	d4	d5	d6	d7	d8	d9	d10	d11	d12	d13	d14
**1**	22.9	21.9	23.4	23.2	23.8	22.8	23.8	23.6	24	23.5	23.6	23.4	23.5	23.8	23.9
**6**	20.8	21.6	21.9	22	22.2	20.9	21.5	21.8	21.5	21.6	21.8	21.4	22	22.6	22.2
**15**	23.8	23.3	23	23.3	23.1	23.2	23.2	23.4	23.6	23.5	23.6	23.6	24.1	24.7	24.7
**21**	22.6	21.5	21.9	21.3	21.7	21.8	22	21.4	22	21.7	21.7	22.1	22.1	22.4	21.9
**38**	24.1	22.9	22.5	23.1	23.3	23.6	23.1	23.3	23.5	23.5	23.8	23.8	23.6	23.4	23.3
**43**	24.1	22.4	21.9	22.4	22.7	22.8	23.8	22.5	23.4	23.5	23.4	23.6	24.2	24.2	23.2
**48**	24.5	22.6	21.8	21.7	21.4	22.8	22.5	22.6	23.4	23.7	24.2	25	25.1	24.8	24
**60**	27.6	25.3	24.9	26	26.5	26.9	27.6	27.3	27.3	27.5	27.5		27.7	27.7	27.7
**79**	26.1	25.4	25.1	25.7	26.2	26.6	26.4	26.7	26.9	27.3	27.1	27.4	28.1	28.5	27.8
**83**	24	22.4	21.7	21.8	22.3	22.4	22.6	22.6	23.5	23.6	23.6	24	24.4	24.5	23.6
**122**	21.8	19.7	19.7	20	20.2	20.6	20.6	20.2	21.1	21.2	21.4	21.1	21.2	21.2	21.2
**136**	22.1	20.8	20.7	21.2	21.3	22	21.8	22.2	22.5	22.1	22.1	22.2	22.4	22	21.8
**138**	22.6	21.2	21.1	21.5	21.6	22.1	21.7	22.2	22.2	22	21.8	22	22.1	22.4	22.1
**147**	23.1	21.7	21.7	22.4	22.5	22.7	22.7	22.6	23.7	23.7	23.3	23.3	23.5	23.5	23.2
**155**	24	22.3	21.9	22.5	23	23	23.4	22.8	23.3	23.8	24.2	24.6	24.2	24.3	23.3
**157**	26.2	24.9	24.5	25	25	25.6	25.6	25.1	26	26.2	26	25.6	25.2	25.9	25.1
**162**	26.3	25	24.3	24.5	24.7	25	25	25.1	25.9	25.5	25.3	24.9	25	25.1	24.3

Sham 14 days

animal n.	pre	d1	d2	d3	d4	d5	d6	d7	d8	d9	d10	d11	d12	d13	d14
**10**	23	23.7	23.8	24.2	23.7	23.8	23.4	23.6	23.4	23.7	23.5	24	24.2	24	24.4
**54**	24.4	24.2	23.6	23.8	24.2	24.6	24.4	23.6	24.7	25.2	25.3	25.4	25.4	25.7	24.9
**56**	26.3	25.7	25.8	26	26.1	26.2	26.9	26.6	26.8	27.2	26.8		27.7	28	27.2
**88**	24.5	22.8	22.3	22.6	23.2	24.1	24.1	23.9	24.6	23.9	24.2	24.7	24.6	24.9	23.7
**135**	22.8	21.5	21.6	21.5	21.5	22.3	23	22.8	23	22.8	22.3	22.5	23	22.7	22.6
**139**	23.3	22.6	22.6	23	23.3	23	23.2	22.5	22.9	23.7	24.1	23.5	23.6	23.8	23.2
