# Supplementary material for: Correction: Transthyretin expression in the postischemic brain
Source: PLoS One. 2020 Jun 25;15(6):e0235527. doi: 10.1371/journal.pone.0235527 (PMC7316325; doi:10.1371/journal.pone.0235527)
Supplement: S2 File — (PDF) [file pone.0235527.s002.pdf]

Temperature (ºC)

PT 24 hours

animal n.	pre	d1
**61**	35.2	35.8
**62**	37.8	37.5
**63**	35.6	35.3
**64**	38.9	37.4
**65**	38.8	37.9
**94**	37.1	37.5
**98**	37.9	36
**99**	36.4	37.1

PT 48 hours

animal n.	pre	d2
**67**	36.4	38
**68**	38.4	38.9
**69**	37.5	36.4
**70**	38.6	37.9
**92**	36.3	38.1
**93**	36.1	37.2
**100**	37.2	37.1
**101**	38	37.5

PT 7 days

animal n.	pre	d7
**76**		
**77**		
**78**		
**109**	36.5	36.6
**110**	38.9	37.1
**111**	38.3	37
**112**	37.9	36.5
**113**	37.9	37.2

Sham 48 hours

animal n.	pre	d2
**102**	36.9	37.1
**103**	38.3	37.1
**104**	39.4	37.6
**105**	36	38
**106**	37.9	36.9

Sham 7 days

animal n.	pre	d7
**107**	38.5	38.2
**108**	38.7	38.1

Temperature (ºC)

PT 14 days

animal n.	pre	d1	d2	d7	d14
**1**	37.7	39.1	37.1	37.6	37.5
**6**	37.4	38.7	38.5	38.5	37.3
**15**	36.3	37.5	38.9	37.3	37.8
**21**	35.9	37.5	37.4	37.8	37.2
**38**	36.4	37.3	37	37.9	37.6
**43**	38.7	37.4	37.1	38.2	39.5
**48**	36.8	37.8	37.3	38	38.4
**60**	37.8	37.7	36.5	38.9	39.1
**79**	38.7	37.3	38.4	39.4	39.1
**83**	37.9	37.4	38.2	38.1	37.8
**122**	36.8	37	37.9	38.9	37.5
**136**	36.5	37.1	38	37.8	37.4
**138**	36.8	37.4	37.4	38	37.4
**147**	37.2	37.6	37.7	37.4	37.9
**155**	37.2	37.6	37.8	37.7	37.4
**157**	37.3	37.6	37.1	38.1	37.8
**162**	36.9	36.7	37.7	38	38.2

Sham 14 days

animal n.	pre	d1	d2	d7	d14
**10**	36.4	38.2	37.8	37.9	37.1
**54**	37.4	36.9	37.3	39.3	39.1
**56**	38.2	37	37.4	38.4	39
**88**	37.9	37.6	37.2	37.8	37.4
**135**	37.3	36.7	37.3	37.7	37.4
**139**	37.3	36.3	38.2	37	37.8
