# Supplementary material for: Correction: Transthyretin expression in the postischemic brain
Source: PLoS One. 2020 Jun 25;15(6):e0235527. doi: 10.1371/journal.pone.0235527 (PMC7316325; doi:10.1371/journal.pone.0235527)
Supplement: S3 File — (PDF) [file pone.0235527.s003.pdf]

qRT-PCR

Positive control

CP	TTR mRNA
**1**	0.595
**2**	3.140
**3**	0.672
**4**	0.932
**5**	0.855

CP - Choroid Plexus

PT 48 hours

animal n.	TTR mRNA
**67**	4.06E-05
**69**	2.35E-03
**68**	9.56E-07

PT 7 days

animal n.	TTR mRNA
**110**	6.75E-07
**109**	2.03E-06
**76**	1.15E-06
**77**	2.50E-05

PT 14 days

animal n.	TTR mRNA
**136**	6.49E-06
**122**	9.49E-05
**42**	5.72E-05

Sham 48 hours

animal n.	TTR mRNA
**102**	1.16E-06
**103**	2.01E-05
**104**	1.51E-05
**105**	8.12E-07
**106**	6.48E-07

Sham 7 days

animal n.	TTR mRNA
**107**	5.77E-07
**108**	9.06E-07

Sham 14 days

animal n.	TTR mRNA
**88**	3.38E-06
**135**	9.98E-06
